# Dexmedetomidine effects on metabolic rate over 5 days in humans

**DOI:** 10.1371/journal.pone.0359711

**Published:** 2026-10-27

**Authors:** Clifton W. Callaway, Alexandra J. Weissman, Katharyn L. Flickinger, Victor Wu, Jenna Monteleone, Ryann DeMaio, Mara Hierro, Daniel Neofes, Omarf Ortega-Reyes, Tao Sheng, Marianna Salinas, Paul Lerro, Francis X. Guyette, Benjamin D. H. Gordon, Marie Mortreux, Kathleen Melanson, Daniel J. Buysee, Philip E. Empey

**Affiliations:** 1 Department of Emergency Medicine, University of Pittsburgh School of Medicine, Pittsburgh, Pennsylvania, United States of America; 2 Department of Health and Human Development, University of Pittsburgh School of Education, Pittsburgh, Pennsylvania, United States of America; 3 Department of Nutrition, University of Rhode Island College of Health Sciences, Kingston, Rhode Island, United States of America; 4 Department of Psychiatry, University of Pittsburgh School of Medicine, Pittsburgh, Pennsylvania, United States of America; 5 Center for Clinical Pharmaceutical Sciences, Department of Pharmacy, University of Pittsburgh School of Pharmacy, Pittsburgh, Pennsylvania, United States of Americaand Therapeutics, University of Pittsburgh School of Pharmacy, Pittsburgh, Pennsylvania, United States of America; Scuola Superiore Sant'Anna, ITALY

## Abstract

The alpha-2-adrenergic agonist drug dexmedetomidine induces sleep-like sedation and reduces global metabolic rate. Drug-induced metabolic suppression sustained over days in humans could be a strategy for reducing energy expenditure (EE), oxygen consumption (VO_2_), and carbon dioxide production (VCO_2_) during prolonged rescue or austere conditions with limited life-support. We tested the feasibility of reducing EE, VO_2_ and VCO_2_ measured with intermittent indirect calorimetry using subcutaneous infusion of dexmedetomidine 1 µg/kg/hour for 18 hours per day over five days in healthy adults. Five participants received multiple days of dexmedetomidine in a laboratory setting, and five separate participants received placebo for one or five days. Relative to resting metabolic rate, dexmedetomidine infusion reduced VO_2_ (effects, 95%CI, at 6-hour time epoch: −0.084 L/min, −0.145 to −0.023), VCO_2_ (−0.067 L/min, −0.130 to −0.005) and EE (−577 kcal/day, −1016 to −137). Relative to placebo, dexmedetomidine produced light sedation (Richmond Agitation Sedation Score: −1.25; 95%CI −1.70 to −0.80 relative to placebo), with lower heart rate (−15 beats/minute; 95%CI −23 to −7) and core body temperature (−1.3ºC; 95%CI −1.7 to −0.8). Dexmedetomidine effects on all physiological variables decreased over time and were absent after day 2. Maximum and minimum plasma drug levels did not change over days. Participants reported rebound anxiety and insomnia after the study. Administering a sedating level of dexmedetomidine that reduces VO_2_, VCO_2_, and EE for 2 days was feasible in healthy adults. However, tolerance to drug effects develops by 3 days, which is a critical limitation for long-term use of this drug regimen.

## Introduction

Controlling metabolic demand is a potential strategy to prolong survival when food or oxygen are limited. Many animals can enter a torpor or hibernation state during winter or starvation, with 70%−95% reductions of energy expenditure [1,2]. Reduced core body temperature is one hallmark of hibernation during which animals turn off the normal cold-induced thermogenesis reflexes that increase metabolic rate as body temperature falls [1]. Humans do not normally display the traits that allow hibernation, but many studies have explored whether features of hibernation could be adapted to humans for medical indications or for extreme activities or environments [3]. Immediate applications might include reducing organ metabolic demand during medical procedures or shock states, extending the time available for rescue or return after catastrophic life-support failure in spaceflight, or reducing oxygen (O_2_) consumption and carbon dioxide (CO_2_) accumulation during prolonged rescue from a closed space such as a submarine or cave [4,5]. Future applications might include intentional hibernation to conserve resources during long-duration spaceflight [6–8].

Anesthetic drugs can reduce metabolic rate in humans [9,10]. However, many of these drugs reduce respiration and require ventilatory support with prolonged use. We and others observed that alpha-2-adrenergic agonists can induce sleep-like sedation, reduce global metabolic rate, and suppress cold-induced thermogenesis, without impairing respiratory drive [11–14]. One such drug, dexmedetomidine, can be delivered parenterally to achieve reliable drug levels and has a short 2- to-3-hour half-life that allows for rapid recovery [15]. We demonstrated previously that subcutaneous dexmedetomidine infusion at 1.0 µg/kg/hour could reduce O_2_ consumption and CO_2_ excretion in healthy human participants without mechanical ventilatory support [15].

It is not known if a drug like dexmedetomidine is practical for prolonged reduction of metabolic demand in humans. Tolerance to alpha-2-adrenergic receptor agonists has been reported, and new side-effects may become apparent with repeated or prolonged administration. As a first step to explore prolonged administration, we conducted a 5-day laboratory study in healthy adult participants. In this study, participants received an 18-hour infusion of drug repeated daily for 5 days, allowing a 6-hour interruption in drug infusion for participants to eat, exercise and self-care. This study tested if prolonged dexmedetomidine infusions in spontaneously breathing, healthy humans can reduce O_2_ consumption, CO_2_ excretion, and energy expenditure (EE) over 5 days.

## Materials and methods

The University of Pittsburgh Human Research Participant Protection Office approved this study, and all participants provided prospective written informed consent. Participants completed research activities between April 2024 and September 2025.

### Study design

We assigned participants to receive either dexmedetomidine or saline placebo for 18 hours per day for up to 5 consecutive days. For safety, neither participants nor investigators were blinded to drug assignment.

The study protocol was designed with technical requirements for application in a target spaceflight mission [6–8]. Specifically, we required (1) minimally invasive drug delivery that could potentially be self-administered, (2) daily sedation interruptions to allow participants to toilet, eat and self-care, and (3) time each day for exercise to maintain muscle health. The first two requirements eliminate the need for catheters, parenteral feeding or assisted hygiene. The third requirement represents the fixed cost of current countermeasures to microgravity-associated muscle loss. We tested 18 hours of drug administration, 5 hours of recovery and 1 hour of supervised exercise as the first version of this protocol (S2 Fig).

### Participants

We recruited healthy persons aged 18–55 years old through advertisements in our university research participant registry (Pitt+Me).

### Screening

Potential participants provided medical history including medication use. We excluded persons with sleep disorders, psychiatric conditions, or medical conditions that might be adversely affected by altered diet and schedules in the laboratory (e.g., diabetes). We excluded persons taking medications that would interact with sleep or sedation (e.g., stimulants or anxiolytics). We measured height, weight and % body fat. We excluded persons with weight<55 kg or Body Mass Index >30. Participants completed the Epworth Sleepiness Scale, and we excluded persons with scores > 10, corresponding to excessive daytime sleepiness. We obtained a 12-lead electrocardiogram, and excluded persons with signs of AV conduction delays, QT prolongation or dysrhythmia. We measured grip strength using a handheld device (JAMAR, Chicago IL, USA). Participants completed a modified Bruce Protocol exercise treadmill test during which we monitored O_2_ consumption (VO_2_) and CO_2_ excretion (VCO_2_) with a metabolic cart (Parvo-medics Inc., Parvo TrueOne 2400, Sandy, UT, USA). We excluded participants with VO_2_ < 20 percentile or >90 percentile of expected values for age and sex according to the ACSM charts [16].

### Study days

We confirmed that participants capable of becoming pregnant had a negative urine pregnancy test at the beginning of all laboratory visits. We placed an intravenous catheter in one arm for blood draws. Participants swallowed a telemetry temperature measuring capsule prior to study start (eCelsius using research mode, BodyCap, Hèrouville Saint-Claire, France). If this capsule passed through the gastrointestinal tract prior to the end of the laboratory study, participants swallowed a replacement capsule. Time zero was defined as the time that participants started drug, which occurred between 10:30 and 12:00 local time.

During drug infusion, participants were confined to the laboratory and allowed to sit or recline on a bed with a temperature-controlled surface set to 22–28ºC based on comfort. A medical water circulation cooling device (Arctic Sun, Franklin Lakes, NJ, USA) was used to control the bed surface temperature. Food and fluids were available ad libitum. Participants could ambulate to the toilet with assistance, or we provided them with a urinal. Urine output was collected in 24-hour blocks. Participants were weighed daily.

### Drug administration

At time zero for each day, participants ingested 2 µg/kg dexmedetomidine or an identical volume (0.02 ml /kg) of saline. Drug was dripped under the tongue, held for 60 seconds and any remainder swallowed. Simultaneously, we started a syringe pump (MedFusion 3500 Syringe Pump, Medex Inc. Duluth, Georgia, USA) to deliver an infusion of dexmedetomidine 1 µg/kg/hour or saline at a rate of 1.5 ml / hour via a subcutaneous needle (Medtronic Minimed 6 mm or 9 mm Quick Set, Northridge, CA, USA) placed in the skin of the lower abdomen. Dexmedetomidine was the intravenous preparation which was diluted with 0.9% saline for the subcutaneous infusion.

We selected drug doses and routes based on our prior studies that measured dexmedetomidine levels after 1–4 µg/kg oral boluses and continuous subcutaneous infusions of 1 µg/kg/hour [14,15]. We selected subcutaneous infusions rather than intravenous infusions as part of the technical requirement for minimally invasive and self-administered drug delivery. We predicted that the regimen in this protocol would achieve sedating levels of drug within 1–2 hours and that participants would be able to participate in testing or exercise about 4 hours after stopping drug.

### Post-drug waking and exercise

Drug infusions stopped after 18 hours. Researchers woke participants at 20 hours. Participants completed a supervised exercise protocol between 23 and 24 hours using a flywheel-based resistance device (kBox, Exxentric North America Inc., Portland, OR) and a handheld isometric exercise device (KINVENT, Montpellier, France) (S1 Table).

### Measurements

We monitored heart rate constantly via a surface patch (ProPatch, Lifeware Inc., Pittsburgh, PA) and deep gastric temperature from the swallowed capsule. We recorded blood pressure from an arm cuff every hour. Researchers recorded the Richmond Agitation Sedation Score (RASS) every 4 hours [17]. RASS score of 0 corresponds to alert and calm, positive scores up to +4 indicate agitation, and negative scores ranges included drowsy (−1, eyes open to voice and remain open>10 seconds), light sedation (−2 eyes open to voice for <10 seconds), deep sedation (−3, movement or eye opening with no eye contact to voice), deep sedation (−4, eyes open or movement only to physical stimulation) and unarousable (−5).

### Indirect calorimetry

We placed a clear plastic canopy over the participant while they were lying supine in the bed to capture exhaled breath and measure VO_2_ and VCO_2_ (Parvo TrueOne 2200 Metabolic Cart, Parvo Medics, Salt Lake, UT, USA) with one minute time resolution. When participants were sleeping, we were able to place the canopy with minimal disturbance other than requiring the participant to remain lying supine (S1 Fig). We obtained a resting metabolic rate (RMR) over one hour on the first day prior to any drug administration. On each day of drug administration, we measured metabolic rate at 6–7 hours (during infusion) and 17–18 hours (just before end of infusion) after drug start. We excluded measurements where the fraction of expired CO_2_ < 0.5%, which corresponded to time when the canopy was not on the participant. At 23 hours after drug start, participants breathed into a facemask to collect VO_2_ and VCO_2_ while sitting quietly for 15 minutes and then continued to wear the facemask during a supervised exercise session between 23 and 24 hours. We excluded measurements where tidal volume was less than 300 ml, which corresponded to times when the mask was leaking or when the participant was trying to talk. Energy expenditure (EE) at each measurement was calculated from the Weir Equation: energy expenditure (kcal/24 hours) = 1440 (3.9 x VO_2_ + 1.1 x VCO_2_) [18].

We calculated the daily total energy expenditure (TEE) by extrapolating the measurements while under the canopy in each condition to the entire 24 hour period. Specifically, we multiplied the hourly EE measured at 6–7 hours (EE_6–7_), 17–18 hours (EE_17–18_) and at 23 hours sitting (EE_sitting_) or exercising (EE_exercise_) by the approximate number of hours in each condition. Dexmedetomidine participants spent 0–20 hours quietly sedated or sleeping (20 hours x average of EE_6–7_ and EE_17–18_), 20–23 hours sitting (3 hours x EE_sitting_) and 23–24 hours exercising (1 hour x EE_exercise_). Placebo participants spent 0–12 hours sitting or reclining in laboratory (12 hours x EE_6–7_), 12–20 hours lying quietly or sleeping (8 hours x EE_17–18_), 20–23 hours sitting (3 hours x EE_sitting_) and 23–24 hours exercising (1 hour x EE_exercise_). To confirm robustness of these results, we also calculated dexmedetomidine TEE using the same formula as used for placebo: 12 x (EE_6–7_) +8 x (EE_17–18_) + 3 x (EE_sitting_) + 1 x (EE_exercise_).

For comparison, we calculated the predicted RMR [19] and the predicted daily total energy expenditure (pTEE) [20] based on anthropometric measurements. We also considered an estimated daily total energy expenditure (eTEE) as 1.35 x the measured RMR.

As an additional measure of metabolic rate, dexmedetomidine participants ingested 1.25 g/kg doubly labeled water (DLW) 5 days prior to the laboratory study. DLW was 10% deuterium oxide (^2^H_2_O) and water with ^18^O (H_2_^18^O), diluted with local drinking water. We obtained plasma samples at baseline, 6–24 hours after ingestion, at the beginning of the laboratory stay, and 4–5 days into the laboratory stay. Using standard calculations [21], we compared the rate of disappearance of ^2^H and ^18^O from total body water. The excess rate of ^18^O disappearance is proportional to CO_2_ excretion and total energy expenditure. For each participant, we calculated the pre-laboratory energy expenditure and the in-laboratory energy expenditure. One placebo participant also provided DLW data for comparison.

### Safety parameters

A physician was present during all drug infusions. When heart rate was persistently below 35 beats per minute or systolic blood pressure below 80 mmHg, a study physician could pause dexmedetomidine infusion for 30 minutes and reevaluate. At that time, study drug could be paused for an additional 30 minutes, restarted or restarted at 0.5 µg/kg/hour. Physicians had option to terminate the drug infusion for any safety concerns. Research staff accompanied participants whenever they were ambulatory to prevent falls. Participants were warned and prepared for orthostatic symptoms, with staff reinforcing caution during transitions from supine to sitting to standing.

### Drug levels

We collected plasma at 18 hours (peak) and 24 hours (trough) after start of drug infusion. Dexmedetomidine levels were measured using LC-MS/MS methods described previously [14,22].

### Statistical analysis

This study was originally designed as a single-arm study in which every participant would receive drug, and drug effect would be quantified by the change in metabolic rate compared to baseline RMR. A target sample of 8 participants was selected to be able to detect a 30% decrease in metabolic rate after drug. After 5 participants received dexmedetomidine, there was clear evidence of tolerance to the drug, and the investigators stopped enrollment given that this new information informed the feasibility of the intervention and altered the risk profile for subsequent participants. We shared the interim findings with and solicited the opinion of participants who had completed screening but not yet participated in the drug phase when deciding to stop early. Placebo participants were added post hoc to clarify any laboratory-specific circadian or sleep-related changes in measures.

To analyze indirect calorimetry data, which was sampled intermittently, we defined five time epochs relative to the start of drug infusion: 0 hours (RMR during resting prior to drug), 6 and 18 hours (during drug or placebo infusion), 23:00–23:15 hours (sitting quietly after drug) and 23:16–24:00 hours (exercise). We created regression models during non-exercise epochs using random intercepts to account for clustering of repeated measurements within participants. The epochs with exercise were analyzed separately because activity level during that epoch was coached by the staff. For DLW data, we described the mean change in total energy expenditure for each participant with 95%CI.

Our planned primary analysis considered a model for energy expenditure, VO_2_, VCO_2_ and other secondary measures fit only to the participants who received dexmedetomidine with epoch and study day and their interaction as independent categorical factors. Drug effect was defined as the change in the physiological measure relative to baseline during epochs with drug infusion (6 or 18 hours). For RASS score, temperature, heart rate, systolic blood pressure and diastolic blood pressure, which were sampled continuously across study days, we also examined regression models using all hourly measurements instead of epochs to confirm the patterns of results.

We repeated these models in placebo participants to determine the pattern of changes in the absence of drug. A separate analysis including all drug and placebo participants considered a model with drug (dexmedetomidine or placebo), epoch, study day and their interaction as independent variables. Drug effect in that model was the defined as the effect of change from placebo to drug.

We compared marginal means from fit models with Bonferroni correction for multiple comparisons. For post-hoc comparison of observed values at each epoch and day versus baseline RMR, we compared means with Dunnett correction for multiple comparisons. After recognizing that drug effect changed over study days, we also contrasted means for observed values between drug and placebo for each epoch on the first study day alone. Values are reported as means with standard deviation (SD) or 95% confidence intervals (95%CI). An alpha-error rate of 0.05 was used to define significant changes. Statistical analysis was completed using Stata v19.5 (StataCorp, College Station, TX, USA) and graphs prepared using Prism v. 11.0 (GraphPad Software, Boston, MA, USA).

## Results

Of 22 persons screened, 10 participated in laboratory stays. Characteristics of the participants are in Table 1. There were no large differences between characteristics of dexmedetomidine and placebo participants. Of these, 5 began and 4 completed a 5-day dexmedetomidine infusion, but one 5-day dexmedetomidine participant had to leave the laboratory early on study day 4 because of a family emergency unrelated to the study. One participant completed a 5-day placebo infusion, and another 4 participants completed a 1-day placebo infusion.

**Table 1:** Characteristics of participants.

N (%) or mean (SD)	Dexmedetomidine (n = 5)	Placebo (n = 5)	Standardized Mean Difference or Odds Ratio (OR) (95%CI)
**Male Sex**	4 (80%)	3 (60%)	OR 0.38 (0.02, 6.3)
**Age in years**	43 (15)	37 (7)	−0.51 (−1.75, 0.77)
**Height in cm**	177 (7)	178 (8)	0.14 (−1.1. 0.14)
**Weight in kg**	73.8 (8.7)	81.0 (13.6)	0.63 (−0.67,1.88)
**BMI**	23.7 (3.2)	25.4 (2.7)	0.58 (−0.71, 1.83)
**VO**_**2**_ **Max in ml/kg/min**	39.0 (5.9)	38.0 (6.5)	−0.17 (−1.41, 1.08)
**Grip score in N**	77 (27)	74 (36)	−0.11 (−1.35, 1.13)
**Resting SBP in mmHg**	133 (12)	125 (12)	−0.72 (−1.98, 0.59)
**Resting DBP in mmHg**	82 (12)	74 (8)	−0.75 (−2.01, 0.56)
**Resting HR in beats/min**	62 (15)	69 (7)	0.58 (−0.71,1.83)
**Predicted RMR (pRMR)**^**a**^ **in kcal/24 hr**	1603 (208)	1677 (270)	0.31 (−0.95,1.55)
**Predicted TEE (pTEE)**^**b**^ **in kcal/24 hr**	2825 (341)	2910 (460)	0.21 (−1.04, 1.45)

^a^ pRMR according to Mifflin St. Jeor 1990 [19].

^b^ pTEE according to Bajunaid 2025 [20].

When comparing the VO_2_, VCO_2_, and EE over the entire 5-day protocol to the baseline metabolic rate determined prior to any drug administration on day 1, during dexmedetomidine infusion, participants had lower marginal VO_2_, VCO_2_, and EE as indicated by the effects, 95%CI, of time epoch: for VO_2_ (6 hour: −0.084 L/min, −0.145 to −0.023; 18 hour −0.072 L/min, −0.133 to −0.011), VCO_2_ (6 hour: −0.067 L/min, −0.130 to −0.005; 18 hour: −0.065 L/min, −0.127 to −0.002) and EE (6 hour: −577 kcal/day, −1016 to −137; 18 hour: −510 kcal/day, −949 to −70) (Fig 1).

**Fig 1:**
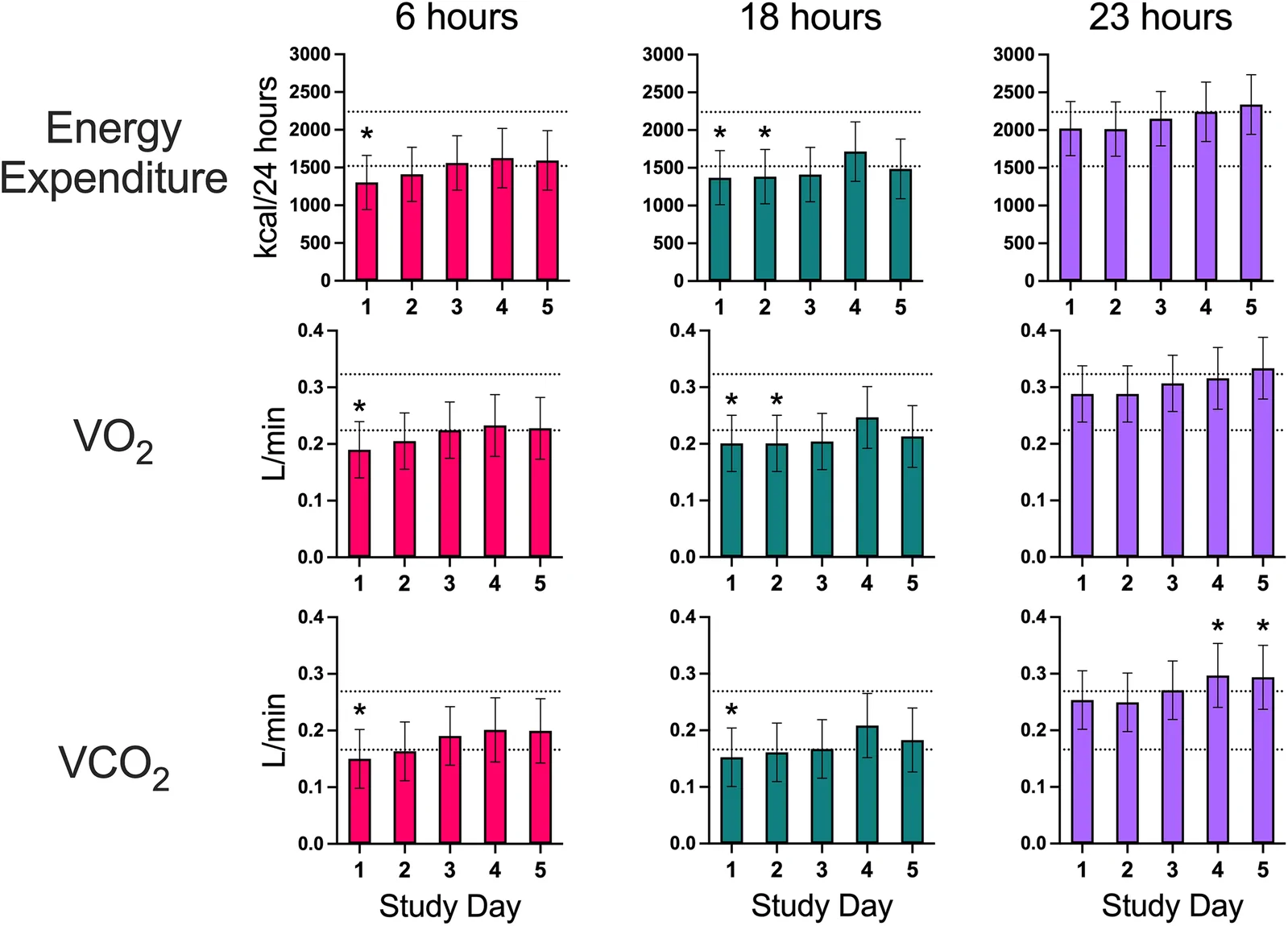
EE, VO2, VCO2 after Dexmedetomidine Compared to Baseline RMR over 5 Days. Mean and 95% confidence intervals for EE (top row), VO_2_ (middle row) and VCO_2_ (bottom row) from indirect calorimetry are plotted for each time point prior to exercise (6, 18 and 23 hours on left, middle and right column respectively) on each study day (1-5). The horizontal lines indicate the 95% confidence intervals for the RMR determined on study day 1 prior to starting drug. Astrik (*) indicates that the measured value differed from baseline RMR, p < 0.05 with Dunnett correction.

In placebo participants, there were no detectable effects of time epoch during placebo infusion for VO_2_, VCO_2_ and EE (S3 Fig).

Dexmedetomidine effects were maximal on the first day of drug infusion (Fig 1). Compared to placebo participants on day 1, dexmedetomidine participants had lower VO_2_ (−28.7%), VCO_2_ (−37.2%), and EE (−30.3%) at 6 hours and lower VCO_2_ (−20.0%) at 18 hours (Table 2).

**Table 2:** Indirect calorimetry measurements on first day.

Mean (SD) or Mean Difference (95% CI)	Resting Metabolic Rate (RMR) (0 hours)	During Infusion (6–7 hours)	End of Infusion (17–18 hours)	Sitting (23 hours)	Exercise (23–24 hours)
**Energy Expenditure (kcal/24 hour)**					
**Dexmedetomidine**	1879 (484)	1303 (156) **	1370 (185) **	2020 (506)	3320 (1398) **
**Placebo**	1737 (433)	1869 (386)	1567 (179)	2152 (422) **	4444 (946) **
**Dexmedetomidine – Placebo**	143 (−527,812)	−566 (−995,-137) *	−197 (−463,69)	−131 (−810,548)	−1124 (−2864,617)
**% Change**	+1.5%	−30.3% *	−12.6%	−6.1%	−25.3%
**VO**_**2**_ **(ml/kg/min)**					
**Dexmedetomidine**	273 (70)	189 (23) **	201 (27) **	288 (72)	466 (189) **
**Placebo**	251 (62)	265 (52)	225 (27)	309 (61) **	629 (134) **
**Dexmedetomidine – Placebo**	22 (−74,118)	−75 (−123,-16) *	−24 (−64,15)	−20 (−118,77)	−163 (−402,76)
**% Change**	+2.0%	−28.7% *	−10.7%	−6.8%	−25.9%
**VCO**_**2**_ **(ml/kg/min)**					
**Dexmedetomidine**	217 (58)	150 (17) **	152 (20) **	254 (64)	445 (213) **
**Placebo**	205 (54)	239 (60)	190 (20)	264 (55) **	575 (128) **
**Dexmedetomidine – Placebo**	12 (−70,94)	−89 (−153,-25) *	−38 (−67,-8) *	−11 (−98,76)	−130 (−386,127)
**% Change**	−2.5%	−37.2% *	−20.0% *	−3.8%	−22.6%

Values are mean (SD) for 5 dexmedetomidine participants and 5 placebo participants

* Placebo different from Dexmedetomidine, mean comparisons p < 0.05

** Epoch Different from Baseline RMR, mean comparisons with Dunnett’s correction, p < 0.05

The dexmedetomidine-related decreases in VO_2_, VCO_2_ and EE diminished over study days (Fig 1), reflected by effects of study day in regressions and by the absence of decreases in VO_2_, VCO_2_ and EE relative to baseline RMR after day 2 (Fig 1). At 6 hours, VO_2_, VCO_2_ and EE were lower than baseline on days 1, but not on days 2, 3,4 or 5. At 18 hours, only VO_2_ and EE were lower than baseline on day 2. None of the VO_2_, VCO_2_ or EE measurements were lower than baseline on days 3,4 or 5. VO_2_ and EE did not differ from baseline RMR during quiet sitting at 23 hours across days, but VCO_2_ was higher at 23 hours on days 4 and 5.

When comparing the VO_2_, VCO_2_, and EE over the entire 5-day protocol to the metabolic rate predicted by anthropometric formulas, dexmedetomidine reduced total energy expenditure but not resting energy expenditure. Predicted RMR for participants (Table 1) was similar to the measured resting energy expenditure at hour 0 on day 1 (Table 3). Over the multiple days of dexmedetomidine, the numerically lower measured total EE (1667 kcal/24 hours, SD 163) was not reliably different than observed RMR (1810 kcal/24 hours, SD 480) or predicted RMR (1603 kcal/24 hours, SD 208). However, the measured total EE was 40.7% lower than predicted total EE and 31.8% lower than estimated total EE (Table 3). In placebo participants confined to the laboratory, measured total EE that was lower than predicted or estimated total EE (−33.9% and −16.9% respectively). The numerically larger decreases after dexmedetomidine were not reliably different from the decreases after placebo (Table 3).

**Table 3:** Daily energy expenditure across all study days.

Mean (SD) in kcal / 24 hour, or Mean Difference (95% CI)	Dexmedetomidine	Placebo	Dexmedetomidine – Placebo	% Change from Placebo
**Mean Energy Expenditure by Time Interval**				
**During Infusion (6–7 hours)**	1495 (197)	1968 (300)	−473 (−711, −234)	−24%*
**End of Infusion (17–18 hours)**	1468 (274)	1689 (192)	−221 (−399, −42)	−13%*
**Sitting (23 hours)**	2147 (572)	2393 (422)	−246 (−631, 139)	−10%
**Exercise (23–24 hours)**	3813 (1400)	4618 (925)	−805 (−1582, −29)	−17%*
**Measured Total Energy Expenditure (mTEE)**				
**Measured Total Energy Expenditure (mTEE)** ^**a**^	1649 (163)	1906 (295)	−257 (−623, 108)	−13%
**Measured TEE vs. Predicted TEE**				
**Predicted TEE (pTEE)** ^**b**^	2825 (341)	2910 (460)	−85 (−684, 515)	−3%
**mTEE - pTEE**	−1176 (−1547, −805)	−1003 (−1220, −787)	−173 (−545, 199)	−17%
**% Change from pTEE**	−42%*	−34%*		
**Measured TEE vs. Estimated TEE**				
**Estimated TEE (eTEE)** ^**c**^	2444 (648)	2294 (579)	149 (−746, 1045)	+6%
**mTEE - eTEE**	−888 (−1591, −184)	−438 (−889, 12)	−450 (−1166, 266)	−103%
**% Change from eTEE**	−36%*	−19%		

For Placebo, values are based on 1 day for four participants and 5 days for one participant (9 total days). For Dexmedetomidine, values are based on 3 days for one participant and 5 days for 4 participants (23 total days).

* Comparison of means, p < 0.05

a For placebo, mTEE = 12 x (6-hour EE) +8 x (18-hour EE) + 3 x (Sitting EE) + 1 x (Exercise EE). For dexmedetomidine, mTEE = 20 x ½ (6-hour EE + 18-hour EE) + 3 x (Sitting EE) + 1 x (Exercise EE)

^b^pTEE according to Bajunaid 2025 [20]

^c^eTEE = RMR x 1.35

Data from DLW for dexmedetomidine indicated a large decrease (−51.8%) from total EE during the 5 days prior to laboratory to days measured in the laboratory (Table 4). The pre-laboratory EE measured with DLW (mean 3193 kcal/24 hours, SD 395) was similar to the predicted total EE based on age, sex, body mass and size (mean 2810 kcal/24 hours, SD 364). For the single placebo participant who spent five full days confined to the laboratory, the decrease in total EE estimated with DLW was smaller (−6.4%).

**Table 4:** Double labeled water estimate of total energy expenditure.

Mean (SD) in kcal / 24 hour, or Mean Difference (95% CI)	Dexmedetomidine (n = 5)	Placebo (n = 1)	Dexmedetomidine – Placebo
**Doubly Labeled Water (DLW) measured TEE**			
**DLW-TEE prior to Lab**	3193 (395)	3829	−636
**DLW-TEE in Lab**	1539 (348)	3584	−2045
**(DLW-TEE)**_**In Lab**_ **–** **(DLW-TEE)**_**Prior to Lab**_	−1654 * (−2131, −1177)	−245	−1409
**% Change**	−52% *	−6%	

* Comparison, p < 0.05

Dexmedetomidine infusion produced light sedation with RASS scores less than zero during all drug-infusion epochs (Fig 2). For the entire day, dexmedetomidine was associated with decreased RASS compared to placebo (drug effect: −1.25; 95%CI −1.70 to −0.80). The mean RASS score for the day was lower after dexmedetomidine than after placebo on the first day but not on subsequent days (Fig 2). During dexmedetomidine infusion, participants were able to wake for toilet needs or to request food and drink. Most reported that they were sleeping or lightly dozing during drug infusion. Placebo participants only slept during local nighttime (approximately protocol hours 12–20).

**Fig 2:**
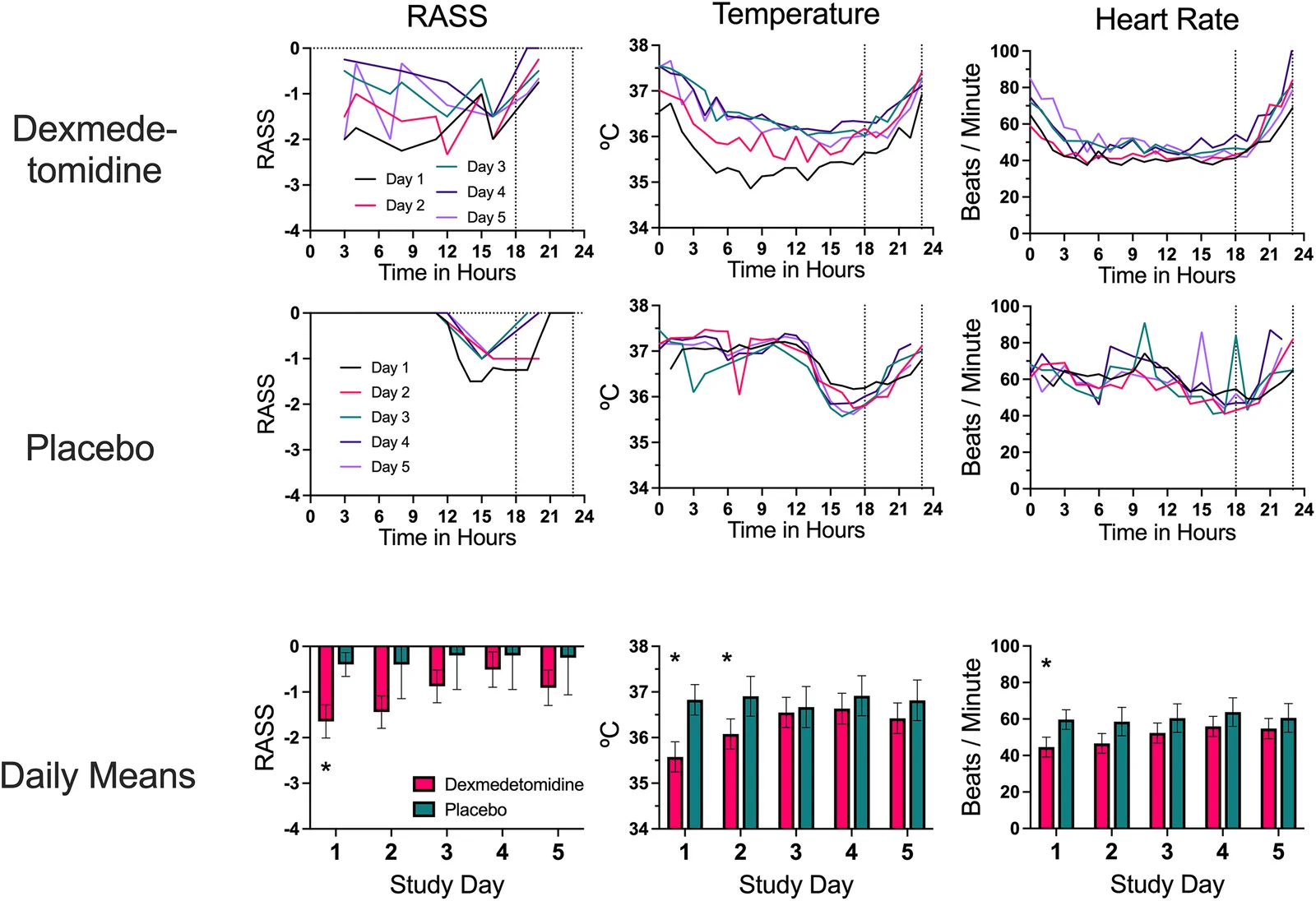
Sedation, temperature and heart rate after dexmedetomidine or placebo over 5 days. Mean hourly values for RASS (left column), temperature (middle column) and heart rate (right column) are plotted with separate lines for each study day (1-5) for the dexmedetomidine participants (top row, n = 5) and for the placebo participants (middle row, n = 5 on Day 1, and n = 1 on subsequent days). RASS was not documented and assumed to be 0 during supervised exercise at hour 23. Marginal daily means for measurements are plotted in the bar graphs (bottom row). Astrik (*) indicates where dexmedetomidine differed from placebo, p < 0.05 with Bonferroni correction.

Dexmedetomidine infusion was associated with a decrease in core body temperature (−1.3ºC; 95%CI −1.7 to −0.8) and heart rate (−15 beats/minute; 95%CI −23 to −7) relative to placebo (Fig 2). Core body temperature showed circadian variation with a decline during sleep in placebo participants. Mean core temperature for the day was lower after dexmedetomidine relative to placebo on days 1 and 2, but not subsequent days. Mean heart rate for the day was lower than placebo on day 1, but not on subsequent days. We did not detect consistent differences between dexmedetomidine and placebo in systolic blood pressure (+5 mmHg; −6–16) or diastolic blood pressure (0 mmHg; −9–8).

Fig 2 shows drug onset over days. Participants reported feeling subjective drug effects by 1 hour after starting infusions. On days 1 and 2, participants were sedated (RASS < −1) by 3 hours and heart rate decreased from 0 to 3 hours. First RASS scores and the numerical values of heart rate were higher on days 3–5. Recovery of heart rate and RASS from 18 to 21 hours did not appear to vary over days.

There was no detectable effect of study day on plasma levels of dexmedetomidine at the end of infusions (mean 1.55 ng/dl, SD 0.55; ß_day_ = −0.045, 95%CI −0.152 to 0.062). (Fig 3) Plasma drug levels declined to mean 0.55 ng/dl (SD 0.38) by 24 hours after drug start (6 hours after drug stop) each day, and these levels also did not change over days of infusion (ß_day_ = −0.063, 95%CI −0.134 to 0.008).

**Fig 3:**
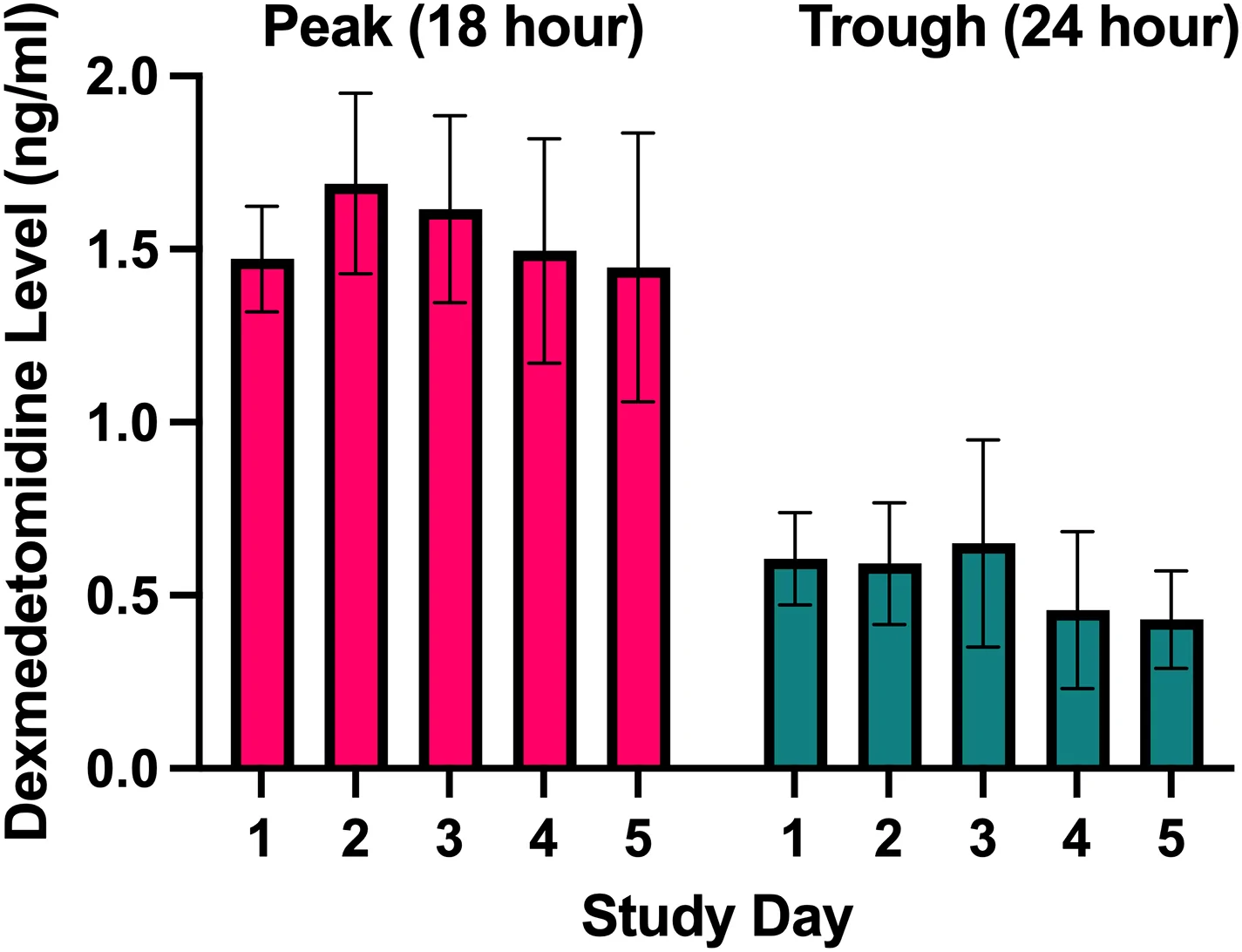
Peak and trough dexmedetomidine levels over 5 days. Mean and 95% CI plasma dexmedetomidine levels (ng/ml) are plotted for each study day (1-5). Peak values were collected at 18 hours at the end of drug infusion. Trough values were collected at 24 hours, which is 6 hours after stopping drug, just before starting next day drug infusion.

Body weight and urine output changed little over days of drug infusion (S2 Table). Blood and urine chemistry had no systematic changes over the course of the study (S2 Table).

There were few adverse events. All dexmedetomidine participants reported orthostatic symptoms, primarily light-headedness or nausea, when standing, which resolved in minutes without treatment or vanished immediately when patients reclined. One participant had persistently low heart rate on the first day of infusion, and the dexmedetomidine dose was reduced to 0.5 µg/kg/hour. This pause was made solely based on the numerical value of the heart rate. The participant had no symptoms or decrease in blood pressure. This participant tolerated the 1 µg/kg/hour dose on subsequent laboratory days. There were no episodes of hypotension or other events requiring treatment during infusions.

All dexmedetomidine participants reported trouble sleeping or anxiety on the first 1–2 days after the laboratory stay. All but one took 0.5 mg – 1.0 mg lorazepam to assist with sleep or anxiety on the first post-study day. One participant sought emergency medical care for presyncope symptoms when he engaged in an athletic event 3 days after the laboratory stay. Vital signs were normal for this participant during that presentation and were attributed to dehydration. All participants reported that restlessness and inability to sleep was worst on the first night after leaving the laboratory. Three reported that sleep duration was normal by the third night after leaving the laboratory, and two did not provide any daily report.

## Discussion

This study demonstrated that administering a sedating level of dexmedetomidine infusion for 18 out of 24 hours for multiple days was feasible in healthy adults. Dexmedetomidine reduced EE during quiet resting by 30.3% and VO_2_ by 28.7% relative to placebo on the first day. Total EE decreased by 42% relative to predicted total EE over the whole session, though simply confining participants to the laboratory reduced total EE by 34% relative to predicted total EE even with placebo administration. Most adverse events were expected and consistent with the known effects of this drug.

While drug effects were robust for at least 2 days in all participants, our data show the development of tolerance to dexmedetomidine after 3 days. The participants reported subjective rebound or withdrawal symptoms for 1–3 days after the infusion. We considered this tolerance to be a critical limiting factor for using this specific drug strategy, and we stopped the study early when this tolerance and rebound became clear. Even in the laboratory, VCO_2_ increased during sitting at 23 hours on days 4 and 5 (Fig 1). Though not statistically different, numerical values for mean VO_2_ and EE were also higher at 23 hours on days 4 and 5, and failure to detect increases in these variables may just be a result of the small number of participants. We suspect that this pattern of increasing metabolic rate at 23 hours is an early sign of the rebound restlessness that participants reported on the post-laboratory days. At a minimum, this pattern indicates that drug effects wore off more quickly and participants were less sedated by 23 hours on days 4 and 5 than they were on prior days.

Tolerance to dexmedetomidine infusion is unlikely to be related to pharmacokinetic changes because peak and trough plasma levels changed little over the 5 days of infusion (Fig 3). The slight increase in peak levels on day 2 probably reflects the accumulation of drug from the previous day rather than a change in volume of distribution. The similar trough levels across all days do not support any increase in clearance. Like other G-protein coupled receptors, alpha-2-adrenergic receptors may undergo phosphorylation or uncoupling within minutes followed by internalization or reduced numbers over hours to days of in response to agonist binding resulting in functional desensitization over time [23,24]. This pharmacodynamic change would explain our results.

Our study provides information about the time-course for developing tolerance to dexmedetomidine in humans, which may be useful for several clinical settings. When dexmedetomidine is used as a sedative, the infusion dose can be titrated to achieve the desired effect. However, our data suggest that a need to increase doses after 2 or more days of infusion may represent tolerance that will lead to withdrawal symptoms when the drug is stopped. Some studies describe protocols for weaning or transitioning to other drugs to prevent dexmedetomidine withdrawal syndrome [25–27].

Using a single dose of drug for all participants ignores between-individual variation in response. In a separate cohort, we observed that a small number of participants were agitated or restless with this dose [15]. At the same time, we had to reduce the dose in some people because of worrisome bradycardia. Our protocol did not allow titration of dose, which is routine when dexmedetomidine is used in medical settings. A protocol in which the drug is titrated to a set sedation level or heart rate might provide different results. To minimize blood draws in this study, we only collected plasma at two time points which does not completely define the drug kinetics. Nevertheless, we did observe some between participant variation in drug levels.

These data suggest that dexmedetomidine reduces EE and VO_2_ during the 5-day protocol both by reducing metabolic rate during rest and by increasing sleep time per day. Dexmedetomidine decreases VO_2_ and EE by about 30% relative to waking (6-hour data in Fig 1) and by about 10% relative to sleeping (18-hour data in Fig 1). At the same time dexmedetomidine increased the total amount of time spent sleeping almost 3-fold from 6–8 hours to 20 hours (RASS data in Fig 2). The combination of lower metabolic rate during sleep and increased sleep time resulted in the large reduction of total EE relative to predicted EE or relative to the EE measured during the prior week using doubly labeled water (Table 3). Some reduction in total EE was evident even after placebo, which reflects the lower total activity when participants were confined to the laboratory compared to their normal daily activity.

Comparison over time between participants receiving dexmedetomidine and participants receiving placebo was a secondary aim of this study that was limited because only one placebo participant spent multiple days in the laboratory. That subject did not show large changes in metabolic rate from day to day, but we cannot exclude the possibility that other participants would have had different patterns over time. The comparison of metabolic rates between placebo and five dexmedetomidine on the first protocol day is based on five participants in each group and more reliable.

The purpose of this investigation was to determine whether a drug-induced decrease in metabolic rate could be maintained for multiple days in healthy humans. Such a reduction would be useful to conserve resources and reduce payload for long-duration spaceflight [6–8] or to improve survivability during long-duration rescues from catastrophic failure of life-support [4,5]. Our results indicate that the current regimen is insufficient for more than 3 days of metabolic suppression. Increasing the dose of drug over time or reducing the daily 6-hour drug interruption might increase the desired effect, but these interventions also would be expected to accelerate drug tolerance. This might worsen the withdrawal symptoms at the end of the study.

Achieving reductions of metabolic rate greater than observed in this study may require other drug classes or larger manipulations of body temperature. We selected an alpha-2-adrenergic agonist for this study because of properties discovered about these drugs during medical use. However, these drugs do not specifically target physiological mechanisms employed by animals that hibernate. Manipulating neuropeptides, adenosine, or endocrine pathways that are implicated in hibernation might better mimic hibernation changes in humans [28,29].

The largest limitation of this study is the small number of participants. The point estimates for metabolic rate and for drug effect on metabolic rate may lack precision. Nevertheless, the sample was sufficient to detect dexmedetomidine-induced reductions in EE and the development of drug tolerance. These data are sufficient to conclude that drug-induced metabolic reduction in humans is technically possible, and that future studies should explore different drugs or dosing regiments for long-term applications.

A second limitation of this study is that we measured baseline EE, VO_2_ and VCO_2_ during the resting state in the laboratory prior to drug administration, which may underestimate the resting metabolic rate that would be observed under stressful situations. Future studies might explore both the resting metabolic rate and any drug effect on metabolic rate in settings that more directly mimic the intended use cases.

This study and related studies in this project [13–15] provide practical lessons for future studies that attempt to mimic animal hibernation and provide a model for how to test metabolic manipulation drugs as they become available. First, short-term experiments can screen for drugs that increase the proportion of total time spent in sleep and also decrease metabolic rate during sleep to levels at or below resting metabolic rate. The short-term experiments also can detect suppression of cold-induced thermogenesis: decreasing core body temperature when heat flux is held constant is one sign of decreased resting metabolic rate. Second, the total energy expenditure is the sum of energy expenditure in all of the behavioral conditions throughout the day: wake, sleep and exercise. Required activities like exercise, eating and toileting have fixed energy costs for any practical implementation. Accounting for all the states during the day is required to determine if a drug provides a net reduction in energy expenditure. Third, there is some reduction in energy expenditure simply from confinement to the laboratory. Studies that compare free living energy expenditure in fit and active adults with drug-states in the laboratory may overestimate the energy reduction attributable to drug unless in-lab control participants are included. Fourth, multi-day studies are required to assess whether tolerance develops to any drug-induced sleep or metabolic rate reduction. The data in this study revealed that participants had less sedation from dexmedetomidine after day 2, which is a serious barrier to implementation. This fact would not have been evident from only short-term laboratory studies.

In conclusion, we demonstrated that dexmedetomidine can be administered to healthy humans without respiratory support for 18 hours/day over 5 days. Tolerance to most physiological effects of dexmedetomidine develops after 2 days, and participants report some rebound symptoms. Nevertheless, this drug regimen does reduce total energy expenditure, O_2_ consumption and CO_2_ production for at least 2 days. This regimen might be useful to prolong survival during emergency situations with limited air or resources.

## Supporting information

S1 Fig Canopy for indirect calorimetry. This picture shows the canopy system used to collect exhaled breath for indirect calorimetry placed over one of the authors in the laboratory in the same configuration used for participants. Participants would lie supine under the canopy during testing for about one hour. When participants were sedated or sleeping, we were able to place the canopy over the participant with minimal disturbance aside from repositioning to supine. Participants could sleep or lie quietly throughout the data collection. For exercise testing at protocol hour 23, we used a traditional face mask to collect exhaled breath.
(TIFF)

S2 Fig Protocol timeline. Timeline for drug administration and testing during daily protocol for A. Dexmedetomidine participants and B. Placebo participants. The daily protocol was repeated each day in dexmedetomidine participants and in one of the placebo participants. Time 0 was the time of drug start, which was typically between 10 am and noon local time. Indirect calorimetry to determine VO_2_, along with VCO_2_ and energy expenditure are indicated as “VO_2_.” Baseline indirect calorimetry was performed prior to drug administration on day 1. Plasma was collected to measure peak drug levels at the end of drug infusion (18 hours) and trough drug levels just prior to starting the next day drug (24 hours). In all subjects, heart rate, blood pressure, sedation scales (RASS), and pulse oximetry were measured during drug infusions and recovery. Dexmedetomidine participants were sedated to quiet resting or sleeping from hours 0–20. Placebo participants were not sedated but quietly resting in the laboratory from hours 0–12, until they went to sleep, typically from hours 12–20. We woke all participants at hour 20 and conducted supervised exercise beginning at hour 23. Indirect calorimetry at hour 23 was collected both while sitting quietly during the first 15 minutes of the supervised exercise, and during exercise.
(TIFF)

S3 Fig Placebo EE, VO2, VCO2 over 5 days. Mean and 95% confidence intervals for EE (top row), VO_2_ (middle row) and VCO_2_ (bottom row) from indirect calorimetry are plotted for each time point prior to exercise (6, 18 and 23 hours on left, middle and right column respectively) for all five participants who received placebo on study day 1 (left column). The horizontal lines indicate the 95% confidence intervals for the RMR determined on study day 1 prior to starting drug. Measured values did not differ from baseline. The individual values from the one participant who received placebo for study days 1–5 are plotted as line graphs (right column).
(TIFF)

S1 Table Exercise protocol. (DOCX)

S2 Table Body weight, oral intake, serum and urine chemistry during dexmedetomidine infusion. (DOCX)
