# Supplementary material for: Dexmedetomidine effects on metabolic rate over 5 days in humans
Source: PLoS One. 2026 Oct 27;21(10):e0359711. doi: 10.1371/journal.pone.0359711 (PMC13712702; doi:10.1371/journal.pone.0359711)
Supplement: Supplementary file 1 [file pone.0359711.s004.docx]

**S1 Table. Exercise Protocol**

Beginning at hour 23 of the protocol, record **i**ndirect calorimetry for 15 minutes while quietly sitting prior to starting exercise. Then perform following exercises while recording indirect calorimetry.

| **Exercise** | **Repetitions** | **Rest Between Sets** | **Sets** | |
| --- | --- | --- | --- | --- |
| **In-Bed Warm-up: Phase I** | | | |  |
| Quadricep Sets | 10 per leg, 5 sec hold per leg | 5 sec between reps | 1 | |
| Straight Leg Lifts (45 degree) | 10 per leg, 5 sec hold per leg | 5 sec between reps | 1 | |
| Hip Abduction Straight Leg | 10 per leg, 5 sec hold per leg | 5 sec between reps | 1 | |
| Hand Iso-ball squeeze | 15 seconds best effort contraction | 15 seconds | 4 | |
| Thigh Iso-ball squeeze | 15 seconds best effort contraction | 15 seconds | 4 | |
| **Standing Warm-up: Phase II** | | | |  |
| Bodyweight Squats | 10 | 45 sec | 2 | |
| Bodyweight calf raises | 10, 5 sec hold at the top of each rep | 45 sec | 2 | |
| Bodyweight good mornings / dead lifts | 10 | 45 sec | 2 | |
| **Flywheel Exercise Protocol** | | | |  |
| Squat (use belt) | 12 | 1 min | 3 | |
| Strict Hold / Static Hold | 10 (4 sec hold, 4 sec off) | 1 min | 3 | |
| Mid-Thigh Pull | 10 | 1 min | 3 | |
| Calf Raises | 8 | 1 min | 4 | |
| **Cool Down** | | | |  |
| Dynamic Stretching / Breath work |  |  |  | |
|  |  |  |  | |
