# Supplementary material for: Dexmedetomidine effects on metabolic rate over 5 days in humans
Source: PLoS One. 2026 Oct 27;21(10):e0359711. doi: 10.1371/journal.pone.0359711 (PMC13712702; doi:10.1371/journal.pone.0359711)
Supplement: Supplementary file 2 [file pone.0359711.s005.docx]

**S2 Table. Body Weight, Oral Intake, Serum and Urine Chemistry during Dexmedetomidine Infusion**

| Mean  (SD) | **Baseline** | **Day 1** | **Day 2** | **Day 3** | **Day 4*** | **Day 5*** |
| --- | --- | --- | --- | --- | --- | --- |
| **Body Weight (kg)** | 71.8  (9.2) | 70.8  (8.9) | 71.9 (8.8) | 72.2 (9.0) | 75.5  (6.1) | 75.7  (8.0) |
| **Total Oral Intake (gm)** | Not measured | 2746.6 (922.0) | 3453.2 (1779.0) | 4263.8 (1901.9) | 3172.6 (1319.0) | 3000.4 (519.1) |
| **Urine Output (ml)** | 2205.0 (1261.0) | 1899.3 (261.5) | 2111.4 (1144.3) | 2619.5 (1013.3) | 2404.5 (880.1) | 1988.2 (773.9) |
| **Urine Calcium (mg/dL)** | 141.9 (290.8) | 5.7 (2.3) | 8.7 (3.2) | 7.8 (5.0) | 10.3 (1.5) | 10.8 (6.5) |
| **Urine Phosphorus (mg/dL)** | 44.1 (34.9) | 37.1 (9.6) | 45.1 (14.4) | 41.5 (20.5) | 53.9 (20.4) | 53.4 (38.3) |
| **Urine Nitrogen (mg/dL)** | 599.0 (452.0) | 409.6 (171.3) | 536.6 (160.9) | 608.0 (216.6) | 636.7 (155.6) | 655.8 (244.3) |
| **Urine Protein (mg/dL)** | 96.2 (93.4) | 80.5 (20.9) | 83.8 (34.1) | 92.2 (63.9) | 89.0 (24.0) | 86.7 (28.7) |
| **Serum Na (mmol/L)** | 138.6 (3.8) | 135.8 (5.3) | 136.4 (4.9) | 137.2 (4.2) | 139.0 (1.6) | 138.5 (2.1) |
| **Serum BUN (mg/dL)** | 14.2 (3.8) | 14.2 (3.3) | 18.8 (2.6) | 20.6 (5.2) | 22.0 (10.7) | 18.0 (5.0) |
| **Serum Creatinine (mg/dL)** | 1.0 (0.2) | 1.0 (0.2) | 1.0 (0.2) | 1.1 (0.3) | 1.1 (0.1) | 1.0 (0.2) |
| **Serum Calcium (mg/dL)** | 9.1 (0.3) | 9.1 (0.5) | 9.2 (0.5) | 9.0 (0.4) | 9.1 (0.4) | 9.2 (0.3) |
| **Serum Phosphorus (mg/dL)** | 3.3 (0.2) | 3.4 (0.6) | 3.5 (0.8) | 3.1 (0.6) | 3.1 (0.4) | 2.9 (0.6) |
| **Serum CPK (IU/L)** | 127.8 (55.5) | 98.6 (67.1) | 114.6 (105.4) | 112.0 (90.0) | 121.5 (71.9) | 96.5 (45.2) |
| **Serum Bilirubin (mg/dL)** | 0.6  (0.1) | 0.7  (0.1) | 0.5  (0.1) | 0.5  (0.1) | 0.4  (0.2) | 0.4  (0.1) |
| **Serum Alkaline Phosphatase (IU/L)** | 47.4  (13.0) | 51.4  (16.0) | 52.2  (14.5) | 49.0  (13.5) | 50.0  (14.1) | 48.2  (13.4) |
| **Serum Aspartate Aminotransferase (IU/L)** | 19.4  (8.0) | 14.2  (4.1) | 15.2  (3.9) | 15.6  (3.6) | 16.2  (7.5) | 16.0  (5.5) |
| **Serum Alanine Aminotransferase (IU/L)** | 16.4  (6.0) | 16.4  (7.1) | 14.8  (3.3) | 15.4  (5.9) | 20.5  (14.7) | 19.8  (13.4) |
| **Serum Prealbumin (mg/dL)** | 27.2  (2.2) | 26.8  (3.8) | 27.4  (3.3) | 25.6  (5.0) | 26.5  (5.4) | 25.0  (5.8) |

* Data not available for one participant on day 4 and 5
