# Supplementary material for: Dexmedetomidine effects on metabolic rate over 5 days in humans
Source: PLoS One. 2026 Oct 27;21(10):e0359711. doi: 10.1371/journal.pone.0359711 (PMC13712702; doi:10.1371/journal.pone.0359711)
Supplement: Supplementary file 3 [file pone.0359711.s006.docx]

7 September 2026

Dear PLOS One:

Thank you for reviewing our manuscript “Dexmedetomidine effects on metabolic rate over 5 days in humans“ for PLOS One. We have considered all of the comments from the peer reviewers and modified our manuscript to address each one. These helpful suggestions have improved the paper, and we hope that you find it suitable now for publication in the journal.

The table below has point-by-point responses to each comment. A marked-up copy of the manuscript is submitted which has track-changes to show where each modification was made.

We also modified fonts and headings according the PLOS One style sheets.

Sincerely,

Clifton Callaway, MD, PhD

| **Reviewer or Editorial Comment** | **Authors’ Response** | **Change in Manuscript** |
| --- | --- | --- |
| ***Editorial Comments:*** |  |  |
| Please ensure that your manuscript meets PLOS ONE's style requirements, including those for file naming. | We made a number of typographical edits to be consistent with the PLOS style sheets. | We reformatted the revised manuscript to match the style. “FIGURE” was changed to “Fig” and “TABLE” to “Table” throughout. Figure legends were moved to be in-line with text. Supplemental information was relabeled “S1 Table,” etc. Headings were formatted per the style sheet. |
| Please note that funding information should not appear in any section or other areas of your manuscript. We will only publish funding information present in the Funding Statement section of the online submission form. Please remove any funding-related text from the manuscript. | We removed the funding information in the Acknowledgements. There are no other acknowledgements, so this section was deleted. | DELETED ACKNOWLEDGEMENTS:  “Acknowledgements. This work was supported by NASA award…” |
| The grant information you provided in the ‘Funding Information’ and ‘Financial Disclosure’ sections do not match…please ensure that you provide the correct grant numbers for the awards you received for your study in the ‘Funding Information’ section. | We correct the funding information to be consistent. | CHANGED FUNDING INFORMATION IN SUBMISSION |
| Please state what role the funders took in the study.  If the funders had no role, please state: "The funders had no role in study design, data collection and analysis, decision to publish, or preparation of the manuscript." | The funders had no role in study design, data collection and analysis, decision to publish, or preparation of the manuscript. | ADDED THIS SENTENCE:  “The funders had no role in study design, data collection and analysis, decision to publish, or preparation of the manuscript.” |
| Please expand the acronym “NIH” (as indicated in your financial disclosure) so that it states the name of your funders in full. | NIH is the National Institutes of Health | CHANGED “NIH” to “National Institutes of Health” |
| We strongly recommend all authors decide on a data sharing plan before acceptance, | We have decided to place the raw data in Zenodo where it can be accessed freely. This plan is consistent with the Open Data policies of our funder. | DATA SHARING PLAN |
| Please upload a copy of Figure 3 | Apologies that this figure was not included in the previous PDF | FIGURE 3 is uploaded to system |
| your supplementary tables are included in the manuscript file. Please remove them and upload them with the file type 'Supporting Information'. | The Supplementary Tables is separated into a different file, now included in the “Supporting information” | MOVED Supplementary Table to the Supporting Information |
| Your supplementary figures are uploaded with the file type 'Figure'. Please amend the file type to 'Supporting Information'. | We revised the file types and names per the PLOS style sheet. | CHANGED the file type for Supplementary Figures to Supporting Information |
|  |  |  |
| ***Reviewer 1:*** |  |  |
| Supplemental Figure. I believe these error bars need to be corrected, | We agree this figure is deceptive: we used the same code as Figure 1 in an attempt to make the figures look alike, which drew error bars from a regression rather than the raw values. i.e. the original figure extrapolated errors from Day 1 data. Since the placebo data is not structured the same as the dexmedetomidine data, this was a mistake.  To correct this, we made an entirely new figure with 95% confidence interval bars calculated directly from the placebo data for Day 1 (n=5 participants) and with a line graph showing the individual values for the one placebo participant who stayed in the lab for 5 days (n=1) | ADDED NEW SUPPLEMENTAL FIGURE 3.  CHANGED SUPPLEMENTAL FIGURE 3 LEGEND:  “S3Fig. Placebo EE, VO2, VCO2 over 5 Days. Mean and 95% confidence intervals for EE (top row), VO2 (middle row) and VCO2 (bottom row) from indirect calorimetry are plotted for each time point prior to exercise (6, 18 and 23 hours on left, middle and right column respectively) on for all five participants who received placebo on each study day 1 (left column). (1-5). The horizontal lines indicate the 95% confidence intervals for the RMR determined on study day 1 prior to starting drug. Measured values did not differ from baseline. The individual values from the one participant who received placebo for study days 1-5 are plotted as line graphs (right column). “ |
| Furthermore, the fact that the placebo group consisted of n=1 from days 2 through 5 significantly limits the reliability of statistical comparisons over time between the two groups. Please clearly state this as a major limitation of the study in the Discussion section. | We agree with this limitation, and emphasize that the primary comparison is from baseline to . The comparison of placebo versus drug on subsequent days was an additional exploratory aspect f the study. | ADD TO DISCUSSION: “Comparison over time between participants receiving dexmedetomidine and participants receiving placebo was a secondary aim of this study that was limited because only one placebo participant spent multiple days in the laboratory. That subject did not show large changes in metabolic rate from day to day, but we cannot exclude the possibility that other participants would have had different patterns over time. The comparison of metabolic rates between placebo and five dexmedetomidine on the first protocol day is based on five participants in each group and more reliable.” |
| Estimated the total energy expenditure over 24 hours by applying the values to the formula in footnote a of Table 3. What is the basis for this formula? Also, why were different formulas used for the DEX group and the placebo group? Isn’t it inappropriate to compare data calculated using different formulas? | Our approach is to multiply the hourly EE rate estimated during each type of activity x the hours spent in that activity. Different formulas for placebo and dexmedetomidine reflect the fact that dexmedetomidine participants were sedated the entire time between 0 and 20 hours, whereas placebo participants were awake while sitting/reclining between 0 and 12 hours and sleeping between 12 and 20 hours. We believe the displayed formulas most accurately represent the actual activity for participants during the protocol and are the best estimates from indirect calorimetry of the total 24 hours EE.  We tried using the same formulas for estimating the total EE in dexmedetomidine and placebo participants, and this provided a similar pattern of results. This is added to the RESULTS | ADDED TO METHODS: “We calculated the daily total energy expenditure (TEE) by extrapolating the measurements while under the canopy in each condition to the entire 24 hours period. Specifically, we multiplied the hourly EE measured at 6-7 hours (EE6-7), 17-18 hours (EE17-18) and at 23 hours sitting (EEsitting) or exercising (EEexercise) by the approximate number of hours in each condition. Dexmedetomidine participants spent 0-20 hours quietly sedated or sleeping (20 hours x average of EE6-7 and EE17-18), 20-23 hours sitting (3 hours x EEsitting) and 23-24 hours exercising (1 hour x EEexercise). Placebo participants spent 0-12 hours sitting or reclining in laboratory (12 hours x EE6-7), 12-20 hours lying quietly or sleeping (8 hours x EE17-18), 20-23 hours sitting (3 hours x EEsitting) and 23-24 hours exercising (1 hour x EEexercise). To confirm robustness of these results, we also calculated dexmedetomidine TEE using the same formula as used for placebo: 12 x (EE6-7) +8 x (EE17-18) + 3 x (EEsitting) + 1 x (EEexercise).” |
| … more specific details in the “Results” section regarding the timeline of these withdrawal symptoms (e.g., on which day they peaked and on which day they completely resolved). | Unfortunately, we did not anticipate the subjective withdrawal symptoms and there was no structured instrument about anxiety or restlessness completed after the participants left the laboratory. Our observations are based on anecdotal reports from each participant, and the questions we did ask about sleep duration and quality. Two participants failed to answer the post-laboratory sleep questions, so these data are incomplete. | ADD TO RESULTS: “Participants reported restlessness and inability to sleep that was worst on the first night after leaving the laboratory. Three reported that sleep duration was normal by the third night after leaving the laboratory, and two did not provide any daily report.” |
| It is unclear whether DEX directly suppresses metabolism or whether the decrease in metabolism is due to reduced patient activity resulting from its sedative effects. Please state the authors’ opinion on this matter | We believe that both sedation and reduced resting metabolic rate contribute to the observed effects. We add this more clearly in the Discussion | CLARIFY IN DISCUSSION: “These data suggest that dexmedetomidine reduces of EE and VO2 during the 5-day protocol results from both reduction of metabolic rate during rest and from increased sleep time per day. Dexmedetomidine decreases VO2 and EE by about 30% relative to waking (6-hour data in FIGURE 1) and by about 10% relative to sleeping (18-hour data in FIGURE 1). At the same time dexmedetomidine increased the total amount of time spent sleeping almost 3-fold from 6-8 hours to 20 hours (RASS data in FIGURE 2).” |
| Table 3 For placebo, values are based on 3 days for one participant and 5 days for 4 participants (23 total days).: Isn't “placebo” a typo for “DEX”? | This is a typo. | CHANGED TABLE 3 FOOTNOTES: “For Dexmedetomidine, values are based on 3 days for one participant and 5 days for 4 participants (23 total days).” |
| Figure 2 Why doesn't the DEX group have RASS data for 23 hours? | At 23-24 hours, all participants were engaged in the supervised exercise, and RASS scores were not recorded, because they should all be RASS=0. The one line for Placebo Day 1 where RASS=0 for hours 23-24 was graphed because values of 0 were entered into the database for one of those participants. | ADD TO FIGURE LEGEND: “RASS was not documented and assumed to be 0 during supervised exercise at hour 23. “ |
| The authors write that they woke the participants after 20 hours, but was the 18-hour calorimetry conducted while the patients were asleep? Is the canopy designed to allow measurements whether the patient is sleeping or exercising? A photo of the product might help readers visualize it. | The reviewer is correct: the canopy was placed over the sleeping participant with effort to minimally disturb the participant. All participants were able to sleep under the canopy.  We added a Figure of one of the investigators lying under the canopy to demonstrate its placement. | ADD TO METHODS: “When participants were sleeping, we were able to place the canopy with minimal disturbance other than requiring the participant to remain lying supine”.  ADD TO SUPPLEMENT:  S1 Fig.  ADD TO FIGURE LEGENDS:  “S1 Fig. This picture shows the canopy system used to collect exhaled breath for indirect calorimetry placed over one of the authors in the laboratory in the same configuration used for participants. Participants would lie supine under the canopy during testing for about one hour. When participants were sedated or sleeping, we were able to place the canopy over the participant with minimal disturbance aside from repositioning to supine. Participants could sleep or lie quietly throughout the data collection. For exercise testing at protocol hour 23, we used a traditional face mask to collect exhaled breath.” |
|  |  |  |
| ***Reviewer 2:*** |  |  |
| Please clarify why subcutaneous application has been used and additional oral intake rather than boli subcutaneously at the beginning. Please provide more information on pharmacokinetics, possibly from your earlier studies. | We selected the current dosing regimen based on prior studies [REFS 14-15] that examined sublingual loading doses of 1-4 µg/kg and subcutaneous infusions of 1 µg/kg/hr.  Subcutaneous bolus doses are a great idea for an alternative regimen, but we have not tested this to date. | ADDED TO METHODS: “We selected drug doses and routes based on our prior studies that measured dexmedetomidine levels after 1-4 µg/kg oral boluses and continuous subcutaneous infusions of 1 µg/kg/hour [14,15]. We selected subcutaneous infusions rather than intravenous infusions as part of the technical requirement for minimally invasive and self-administered drug delivery. We predicted that the regimen in this protocol would achieve sedating levels of drug within 1-2 hours and that participants would be able to participate in testing or exercise about 4 hours after stopping drug..” |
| Did you screen for other medical conditions, e.g. liver cirrhosis, that can potentially influence the effect of dexmedetomidine? Despite there do not seem to be any group differences for baseline characteristics, please provide an appropriate statistic (Table 1) and add measurement units. | We recruited participants who self-reported themselves to be healthy, and who reported no other organ problems on the PAR-Q survey. After inclusion in the study, we obtained the baseline laboratory data in the Supplemental Table, which suggests no major laboratory abnormality. Specifically, there were no elevations of creatinine to suggest renal dysfunction or transaminases, canalicular enzymes, or bilirubin to suggest liver dysfunction. | ADDED TO SUPPLEMENTAL TABLE: AST, ALT, Alk Phos, Bilirubin, Prealbumin.  ADDED TO TABLE 1: Standardized mean differences for continuous variables and odds ratios for binary variable (sex).  ADDED TO RESULTS: “There were no large differences between characteristics of dexmedetomidine and placebo participants.” |
| Do you have any data if dexmedetomidine has the same effect in these exceptional situations compared to “normal” laboratory days where even “placebo” people have a lower resting rates? | We agree that the effect of the sedative drug might be more pronounced when participants are in stressful situations. The resting state in the laboratory may underestimate the baseline EE, VO_2_ and VCO_2_ prior to drug in those settings, but we are not aware of data directly addressing this. | ADDED TO DISCUSSION: “We measured baseline EE, VO_2_ and VCO_2_ during the resting state in the laboratory prior to drug administration, which may underestimate the resting metabolic rate that would be observed under stressful situations. Future studies might explore both the resting metabolic rate and any drug effect on metabolic rate in settings that more directly mimic the intended use cases” |
| However, if it is supposed to be used for a definite short amount of time, e.g. three days, would it make sense to increase dosage to maintain the goal of “hibernation”? Where would such scenarios of an infusion of sedating substances make sense (outside a defined ICU setting, which is not the aim of this study)? Please clarify what situations you aim for? |  | ADDED TO THE METHODS (mentioned above): “The technical requirements…”  ADD TO THE DISCUSSION:  “The purpose of this investigation was to determine whether a drug-induced decrease in metabolic rate could be maintained for multiple days in healthy humans. Such a reduction would be useful to conserve resources and reduce payload for long-duration spaceflight [6-8] or to improve survivability during long-duration rescues from catastrophic failure of life-support [4-5]. Our results indicate that the current regimen is insufficient for more than 3 days of metabolic suppression. Increasing the dose of drug over time or reducing the daily 6-hour drug interruption might increase the desired effect, but these interventions also would be expected to accelerate drug tolerance. This might worsen the withdrawal symptoms at the end of the study.” |
| State more clearly where you compare what timepoints and which groups. For example, it is hard to follow for the description of the results of Figure 1. Is the marginal reduction of VO2 relative to baseline RMR for day 1 or for all 5 days of follow-up (e.g. effects, 95%CI, of time epoch: 6 hour: -0.084 L/min, -0.145 to -0.023; 18 hour -0.072 L/min, -0.133 to -0.011)? | We added more clear statements at the opening of each paragraph of the Results to declare which comparisons the numbers describe. | ADD TO RESULTS:  “When comparing the VO2 , VCO2 , and EE over the entire 5-day protocol to the baseline metabolic rate determined prior to any drug administration on day 1,…”.  AND  “Compared Relative to placebo participants on day 1, dexmedetomidine participants had lower VO2 (-28.7%), VCO2 (-37.2%), and EE (-30.3%) at 6 hours and lower VCO2 (-20.0%) at 18 hours (TABLE 2). “  AND  “When comparing the VO2 , VCO2 , and EE over the entire 5-day protocol to the metabolic rate predicted by anthropometric formulas, dexmedetomidine reduced total energy expenditure but not resting energy expenditure. “ |
| Why did you apply dexmedetomidine for 18 hours and then pause for 6 hours and did not use any other time frames. Why was dexmedetomidine not infused continuously over five days without any breaks to test if metabolic rates could be reduced. | This regimen was our first proof-of-concept test of metabolic suppression for multiple days in humans. We added to the methods the rationale for the design we selected. | ADDED TO METHODS: “The study protocol was designed with technical requirements for application in a target spaceflight mission [6-8]. Specifically, we required (1) minimally invasive drug delivery that could potentially be self-administered, (2) daily sedation interruptions to allow participants to toilet, eat and self-care, and (3) time each day for exercise to maintain muscle health. The first two requirements eliminate the need for catheters, parenteral feeding or assisted hygiene. The third requirement represents the fixed cost of current countermeasures to microgravity-associated muscle loss. We tested 18 hours of drug administration, 5 hours of recovery and 1 hour of supervised exercise as the first version of this protocol.”  AND  “We selected drug doses and routes based on our prior studies that measured dexmedetomidine levels after 1-4 µg/kg oral boluses and continuous subcutaneous infusions of 1 µg/kg/hour [14,15]. We selected subcutaneous infusions rather than intravenous infusions as part of the technical requirement for minimally invasive and self-administered drug delivery. We predicted that the regimen in this protocol would achieve sedating levels of drug within 1-2 hours and that participants would be able to participate in testing or exercise about 4 hours after stopping drug.” |
| People have a rebound effect of about 2-3 days. How long does it take until you see an effect of the drug when starting the daily experiments and how long does it take until the drug wears off (based on clinical observations such as RAAS)? Does this change over the course of days? I |  | ADDED TO RESULTS: “Figure 2 shows drug onset over days. Participants reported feeling subjective drug effects by 1 hour after starting infusions. On days 1 and 2, participants were sedated (RASS<-1) by 3 hours and heart rate decreased from 0 to 3 hours. First RASS scores and the numerical values of heart rate were higher on days 3-5. Recovery of heart rate and RASS from 18 to 21 hours did not appear to vary over days.” |
| Please provide an overview figure for the setup and the different timepoints. When does infusion start? When does it stop? When do people do exercise, etc. | We added a figure of the study timeline and measurements. | ADDED SUPLEMENTAL FIGURE 2: Study Timeline  ADDED FIGURE LEGEND:  “Supplemental Figure 2. Timeline for drug administration and testing during daily protocol for A. Dexmedetomidine participants and B. Placebo participants. The daily protocol was repeated each day in dexmedetomidine participants and in one of the placebo participants. Time 0 was the time of drug start, which was typically between 10 am and noon local time. Indirect calorimetry to determine VO2, along with VCO2 and energy expenditure are indicated as “VO2.” Baseline indirect calorimetry was performed prior to drug administration on day 1. Plasma was collected to measure peak drug levels at the end of drug infusion (18 hours) and trough drug levels just prior to starting the next day drug (24 hours). In all subjects, heart rate, blood pressure, sedation scales (RASS), and pulse oximetry were measured during drug infusions and recovery. Dexmedetomidine participants were sedated to quiet resting or sleeping from hours 0 to 20. Placebo participants were not sedated but quietly resting in the laboratory from hours 0-12, until they went to sleep, typically from hours 12-20. We woke all participants at hour 20 and conducted supervised exercise beginning at hour 23. Indirect calorimetry at hour 23 was collected both while sitting quietly during the first 15 minutes of the supervised exercise, and during exercise.” |
| How often did the infusion need to be paused due to side effects? Is it only the one patient where infusion had to be reduced at the first day? Was the patient clinically symptomatic due to the bradycardia? | Only one participant required infusion to be paused and reduced because of bradycardia. This pause was made solely based on the numerical value of the heart rate, which we chose a priori as a safety measure as described in the Methods. The participant had no symptoms or decrease in blood pressure. | ADD TO RESULTS:  “This pause was made solely based on the numerical value of the heart rate. The participant had no symptoms or decrease in blood pressure.“  and  “There were no episodes of hypotension or other events requiring treatment during infusions.” |
| Why was only one patient of the placebo group followed up over five days (and the others only one day)? | The primary design of this study was a single-arm comparison of baseline versus drug in the five participants. The additional placebo patients were enrolled as an add-on to explore how the laboratory setting alone affected the metabolic measurements. Unfortunately, we did not have funding for 5-day monitoring of all placebo participants. The one placebo participant who stayed for 5 days showed little day to day variation in measurements, suggesting to us that the one-day observations in the other placebo participants were representative. | DESCRIBED IN METHODS:  “This study was originally designed as a single-arm study in which every participant would receive drug, and drug effect would be quantified by the change in metabolic rate compared to baseline RMR. “  AND  “Placebo participants were added post hoc to clarify any laboratory-specific circadian or sleep-related changes in measures.” |
| Why was VCO2 increased after 23 hours at the last two days and not during the earlier days? Do you have any explanation why EE was not influenced? | This difference is likely a result of our small sample size. All of the variables, EE, VO2 and VCO2 were numerically higher on days 4 and 5 at 23 hours with mean values at or above the upper 95% confidence interval for the baseline values (dashed lines in Figure 1). Only the VCO2 increase achieved statistical significance on days 4 and 5 because the VCO2 values in the laboratory during sitting at 23 hours were already closer to the upper 95% confidence interval of the baseline VCO2. EE and VO2 values during sitting were closer to the mean baseline values. We suspect a larger sample size would have statistically confirmed the EE and VO2 increase.  We suspect that this increase in VCO2 (and the trend to an increase in EE and VO2) is an early sign of the rebound restlessness and sleep disruption that participants reported on the post-laboratory days. At a minimum, this pattern indicates that drug effects wore off more quickly and participants were less sedated by 23 hours on days 4 and 5 than they were on prior days. | ADDED TO DISCUSSION: “Even in the laboratory, VCO2 increased during sitting at 23 hours on days 4 and 5 (Figure 1). Though not statistically different, numerical values for mean VO2 and EE were also higher at 23 hours on days 4 and 5, and failure to detect increases in these variables may just be a result of the small number of participants. We suspect that this pattern of increasing metabolic rate at 23 hours is an early sign of the rebound restlessness that participants reported on the post-laboratory days. At a minimum, this pattern indicates that drug effects wore off more quickly and participants were less sedated by 23 hours on days 4 and 5 than they were on prior days.” |
| In the results section it is stated that VCO2 is increased after 18 hours for the last two days (instead of 23 hours as shown in Figure 1). | The reviewer is correct that this is a mistake in our written description of FIGURE 1: the figure is correct. We correct our written description. | CHANGE RESULTS: “VO2 and EE did not differ from baseline RMR during quiet sitting at ~~18~~ 23 hours across days, but VCO2 was higher at ~~18~~ 23 hours on days 4 and 5.” |
| Table 2: Include p-values and what statistical tests were used |  | ADDED TO Table 2 footnotes:  “* Placebo different from Dexmedetomidine, mean comparisons p<0.05  ** Epoch Different from Baseline RMR, mean comparisons with Dunnett’s correction, p<0.05” |
| Please clarify why measured and predicted PMR was used and what the differences are. |  | Table 3  ADD TO RESULTS:  “When comparing the VO2 , VCO2 , and EE over the entire 5-day protocol to the metabolic rate predicted by anthropometric formulas, dexmedetomidine reduced total energy expenditure but not resting energy expenditure. “ |
| Considering Table 4: Please mark for transparency reasons that the placebo group includes only one person at this point. | For transparency, we add the number of participants in each group to the column headers in Table 4. | TABLE 4. We add numbers to the headers for the column “Dexmedetomidine (n=5)” and “Placebo (n=1)” |
| Unfortunately, Figure 3 is not included | We apologize this was not included in the original submission, and confirm that it is now correctly uploaded. | FIGURE 3 is now uploaded into the editorial system. |
| “5 completed a 5-day dexmedetomidine infusions and 1 participant completed a 5-day placebo infusion 5 completed 5 days of Dexdor, one had a family emergency and left one day early”. Therefore, it does not seem like 5 participants completed the 5 day infusions when 1 participant dropped out early. | The reviewer is correct: only 4/5 completed the 5 days of dexmedetomidine. We clarify this in the text. | RESULTS FIRST PARAGAPH CHANGE TO:” Of these, 5 began and 4 completed a 5-day dexmedetomidine infusion, but one 5-day dexmedetomidine participant had to leave the laboratory early on study day 4 because of a family emergency unrelated to the study. One participant completed a 5-day placebo infusion, and another 4 participants completed a 1-day placebo infusion.” |
| Double-check spelling and grammar throughout the manuscript | Acknowledged. | Spelling and grammar checked. |
| How can you state that lower food intake was seen after dexmedetomidine infusion when no baseline was assessed? Was this compared to placebo? Or just a clinical observation | We did collect food diaries and appetite scales during a day in the week prior to the laboratory stay and during the laboratory stay. These data are complex to interpret because individuals varied greatly in usual diet and because both nausea and sleep affected reporting and eating. These data do not add directly to the current analysis of metabolic rate, and a full analysis and discussion would require a lot of space and be a distraction from the current paper. Therefore, we will save these observations as the topic of another report. | DELETED FROM RESULTS: “Participants had reduced appetite and food intake while receiving dexmedetomidine. “ |
| Please specify your exercise protocol, i.e. as supplemental material. | We developed an exercise protocol that covered all muscle groups using a flywheel device and isometric device that have been used in spaceflight. We attach that protocol in the Supplemental Material and call it out in the Methods. | ADDED TO METHODS: “Participants completed a supervised exercise protocol between 23 and 24 hours using a flywheel-based resistance device (kBox, Exxentric North America Inc., Portland, OR) and a handheld isometric exercise device (KINVENT, Montpellier, France) **(S1 Table).”**  ADDED TO SUPPLEMENTAL MATERIAL: S1 Table. Exercise regimen |
| Please state limitations more clearly, and add that this is a small sample size and data is therefore, mainly hypothesis-generating | We added additional text to acknowledge the limitations. | ADDED TO DISCUSSION: “The largest limitation of this study is the small number of participants. The point estimates for metabolic rate and for drug effect on metabolic rate may lack precision. Nevertheless, the sample was sufficient to detect dexmedetomidine-induced reductions in EE and the development of drug tolerance. These data are sufficient to conclude that drug-induced metabolic reduction in humans is possible, and that future studies should explore different drugs or dosing regiments for long-term applications.  A second limitation of this study is that we measured baseline EE, VO2 and VCO2 during the resting state in the laboratory prior to drug administration, which may underestimate the resting metabolic rate that would be observed under stressful situations. Future studies might explore both the resting metabolic rate and any drug effect on metabolic rate in settings that more directly mimic the intended use cases.” |
